# The factors affecting the survivability of malignant cancer patients with deep vein thrombosis among subjects with gynecologic and non-gynecologic cancer: An ambispective cohort study

**DOI:** 10.12688/f1000research.135252.1

**Published:** 2023-07-26

**Authors:** Andhika Rachman, Griskalia Christine, Rachelle Betsy, Samuel Juanputra, Widya Pratiwi

**Affiliations:** 1 Division of Hematology and Oncology, Department of Internal Medicine, Dr. Cipto Mangunkusumo National Referral Hospital, Faculty of Medicine, Universitas Indonesia, Central Jakarta, Jakarta, 10430, Indonesia; 2 Division of Hematology and Oncology, Department of Internal Medicine, Tarakan Regional Hospital, Central Jakarta, DKI Jakarta, 10150, Indonesia; 3 Department of Internal Medicine, Dr. Cipto Mangunkusumo General Hospital - Faculty of Medicine Universitas Indonesia, Central Jakarta, DKI Jakarta, 10430, Indonesia

**Keywords:** cancer, gynecologic, deep vein thrombosis, survival

## Abstract

**Background:** Gynecologic cancer is a significant public health concern worldwide, with three of the top ten most common cancers affecting women. The increasing incidence of deep vein thrombosis (DVT) and the disproportionately poor outcomes in cancer patients necessitates urgent intervention. This study aimed to analyze the factors affecting the survivability of cancer patients with DVT, especially among gynecologic and non-gynecologic cancers.

**Methods:** An ambispective cohort study was conducted among gynecologic and non-gynecologic cancer patients with DVT, from January 2011 until August 2013.

**Results:** Among 223 cancer subjects with DVT, 61.4% of the subjects developed short-term mortality. In the overall group, the survival time was significantly lower in subjects who developed immobilization status (p-value <0.001), advanced cancer stages (p-value <0.045), and infection status (p-value <0.001). In the gynecologic cancer group, the survival time was significantly lower in subjects who developed immobilization (p-value 0.007) and infection status (p-value 0.021). In the non-gynecologic cancer group, the survival time was significantly lower in subjects who developed immobilization (p-value 0.008), infection (p-value 0.002), undergo cancer surgery (p-value 0.024), and received high-risk systemic therapy (p-value 0.048). Additionally, the most common infection was pneumonia (29.6%).

**Conclusions:** Both gynecologic and non-gynecologic cancer patients who experienced DVT developed a high short-term mortality. Our finding of immobility, infection, advanced cancer stages, systemic therapy, and cancer surgery as risk factors that affect the survivability highlights the necessity of administering secondary prophylaxis as a standard procedure in clinical practice.

## Introduction

Gynecologic cancer is a major threat to global health and comprises three of the top ten most prevalent cancers affecting women worldwide. ^1^^,^^2^ Ovarian cancer is considered to be the deadliest gynecologic malignancy, with the highest mortality rate and the worst prognosis when compared to other types of gynecologic cancers. ^2^^–^^6^ Cervical cancer ranks fourth in terms of both incidence and mortality in women worldwide. In countries with a lower Human Development Index (HDI), cervical cancer is the second most prevalent cancer and cause of mortality, following breast cancer. Nonetheless, it is the most frequently diagnosed cancer in 28 countries and the primary cause of cancer-related deaths in 42 countries. The majority of these countries are located in Southeast Asia and Sub-Saharan Africa. ^7^ After cervical cancer, endometrial cancer is the second most frequent malignancy in developing countries. ^2^^–^^4^^,^^8^^,^^9^

Gynecologic cancer is a challenging and complex disease that poses significant diagnostic and management difficulties. ^10^ It has been found to be underdiagnosed and undertreated, leading to a remarkable 85% of worldwide deaths in developing countries. Furthermore, low-income and middle-income countries have been significantly impacted, with death rates 18 times higher than those observed in wealthier countries. ^7^^,^^10^ For these countries, the massive economic impact posed by lost years of productivity and cancer-related deaths is an extensive challenge. ^2^^–^^4^^,^^11^^,^^12^

Over the past two decades, the association of malignancy with thrombosis events has been established by a significant number of epidemiological studies. ^13^ The incidence of venous thromboembolism (VTE) in patients with active malignancy is four to seven times higher than in the healthy population, ensuring malignancy is one of the most prevalent and significant acquired risk factors for deep vein thrombosis (DVT). ^14^^–^^16^ DVT is a generally treatable condition that poses a significant threat to patients with malignancies. In fact, it is the second-greatest cause of death and has been found to be correlated with reduced survival rates in cancer patients. ^14^^,^^17^ The rising incidence of cancer-associated thrombosis poses an increasing burden on the healthcare system due to the high mortality rate and treatment costs. ^18^

The increasing burden of DVT in cancer patients, along with the disproportionately poor outcomes, requires urgent attention. ^18^ The survival rate is a critical factor in determining the effectiveness of cancer prevention and treatment approaches. ^19^ However, factors affecting the survivability of patients with venous thromboembolism, especially malignancies with DVT, remain unclear. ^20^^–^^22^ To the best of our knowledge, this was the first study conducted to determine the factors affecting the survival time of cancer patients with DVT, specifically among gynecologic and non-gynecologic cancers. Understanding the impact of these factors is essential for establishing targeted preventive and treatment strategies for this specific patient population.

## Methods

### Study design

This was an ambispective cohort study (combination of prospective and retrospective methods) conducted at Dr. Cipto Mangunkusumo General Hospital (CMGH), Jakarta, Indonesia. Data were collected from January 2011 until August 2013. On the prospective method, we collected samples of cancer patients who had DVT at the Division of Hematology and Medical Oncology of CMGH. Retrospective data were extracted from the medical records of CMGH. All subjects were observed for 3 months. The included subjects were aged ≥ 18 years old; subjects with active cancers; subjects with DVT that were confirmed with Doppler ultrasound. Active cancer was defined as newly diagnosed cancer, up to 3 months before DVT diagnosis, or cancer that is being treated. ^23^^,^^24^ Subjects were excluded if they were not available for a 3-month of follow-up (for prospective), or if the medical record data was incomplete (for retrospective).

### Statistical analysis

Extracted data were analyzed with Statistical Package for the Social Sciences (SPSS) version 27 for Macintosh. Any graph or plot was created using GraphPad Prism 9 for Macintosh. The analysis was conducted with Kaplan-Meier survival analysis. The significance was measured using log-rank/Mantel-Cox test.

### Measurement

D-dimer levels were measured by ***immunometric flowthrough*** sandwich ELISA ( ***Nycocard Reader II***). According to the assay manufacturer, the D-dimer cut-off values for DVT was ≥ 300 ng/mL. ^25^

### Ethical approval

Ethical approval for this study was granted by The Ethics Committee of The Faculty of Medicine, Universitas Indonesia (ethical approval number: 495/H2.F1/ETIK/2013). This research was performed in accordance with the Declaration of Helsinki. Prior to recruitment, written informed consent was obtained from the subjects.

## Results

This cohort study included 223 cancer subjects with deep vein thrombosis (DVT). From the 223 recruited subjects, 76.2% were female. Approximately 48.4% of the subjects were diagnosed with gynecologic cancer, which included ovarian, cervical, and endometrial cancers. Non-gynecologic cancers, including lymphoma, leukemia, hepatoma, and breast cancer were found in 51.6% of the subjects ( Table 1).

**Table 1.** Subject characteristics.

Characteristics	N (%)
Age	
<60 years old	181 (81.2)
>60 years old	42 (18.8)
Sex	
Male	53 (23.8)
Female	170 (76.2)
Cancer stages	
Early (I-II)	51 (22.9)
Late (III-IV)	172 (77.1)
Cancer types	
Gynecologic cancers	108 (48.4)
Ovarian cancer	40 (17.9)
Cervical cancer	69 (30.9)
Endometrial cancer	6 (2.7)
Non-gynecologic cancers	115 (51.6)
Lung cancer	7 (3.1)
Hepatoma	11 (4.9)
Hodgkin Lymphoma	17 (7.6)
Rectal cancer	7 (3.1)
Colon cancer	6 (2.7)
Thymoma	3 (1.3)
Breast cancer	19 (8.5)
Mediastinal tumor	3 (1.3)
Nasopharyngeal cancer	5 (2.2)
Laryngeal cancer	1 (0.4)
Osteosarcoma	2 (0.9)
Prostate cancer	4 (1.8)
Intracranial cancer	5 (2.2)
Bladder cancer	3 (1.3)
Pancreatic cancer	1 (0.4)
Leukemia	7 (3.1)
Multiple myeloma	2 (0.9)
Ewing sarcoma	2 (0.9)
Mandibular cancer	1 (0.4)
Perianal cancer	1 (0.4)
Renal cancer	1 (0.4)
The risk of systemic therapy for developing DVT	
Low risk	116 (52.1)
High risk	107 (47.9)
Immobilization > 3 days	
No	53 (23.8)
Yes	170 (76.2)
Central vein catheterization	
No	129 (57.8)
Yes	94 (42.2)
D-Dimer level upon DVT diagnosis	
<300 ng/mL	19 (8.5)
≥ 300 ng/mL	204 (91.5)
Wells score for DVT	
<3	7 (3.1)
≥ 3	216 (96.9)
3-month survivability	
Alive	86 (38.6)
Deceased	137 (61.4)

Abbreviation: DVT, deep vein thrombosis.

Subjects who received tamoxifen, aromatase inhibitors, thalidomide, lenalidomide, bevacizumab, cisplatin, nitrogen mustard, or anthracycline were considered as high-risk systemic therapy for developing DVT. ^1^^,^^26^^–^^41^ Subjects who did not receive high-risk systemic therapy other than those mentioned above were considered low-risk for developing DVT ( Table 1).

Wells score has been utilized for over a decade and has predictive value in determining DVT risk in patients who are hospitalized. ^22^^,^^42^ We divided the Wells score into two categories: subjects with <3 points were considered to have a low probability of developing DVT, whereas subjects with ≥3 points were considered to have a high probability of developing DVT ( Table 1).

Infection was found in 126 patients (56.5%). The most common infections were pneumonia (29.6%), urinary tract infections (19.3%), and chronic viral hepatitis (18.8%). Most of the infected patients had one source of infection (30.5%) ( Table 2).

**Table 2.** Characteristic of infection.

Infection	N (%)
Source of Infection	
BSI	1 (0.4%)
Chronic viral hepatitis	42 (18.8%)
Decubitus ulcer	13 (5.8%)
Intraabdominal infection	7 (3.1%)
Pulmonary tuberculosis	10 (4.5%)
Osteomyelitis	1 (0.4%)
Pneumonia	66 (29.6%)
Tumor mass infection	11 (4.9%)
Urinary tract infection	43 (19.3%)
Number of infection sources	
1	68 (30.5)
2	48 (21.5%)
3	10 (4.5)

Abbreviation: BSI, Bloodstream infection; UTI, urinary tract infection.

In Figure 1A, the survival time was significantly lower in subjects who developed immobilization status compared to subjects without immobilization status in the overall groups (p-value <0.001), the gynecologic cancer group (p-value 0.007), and the non-gynecologic cancer group (p-value 0.008).

**Figure 1A.**
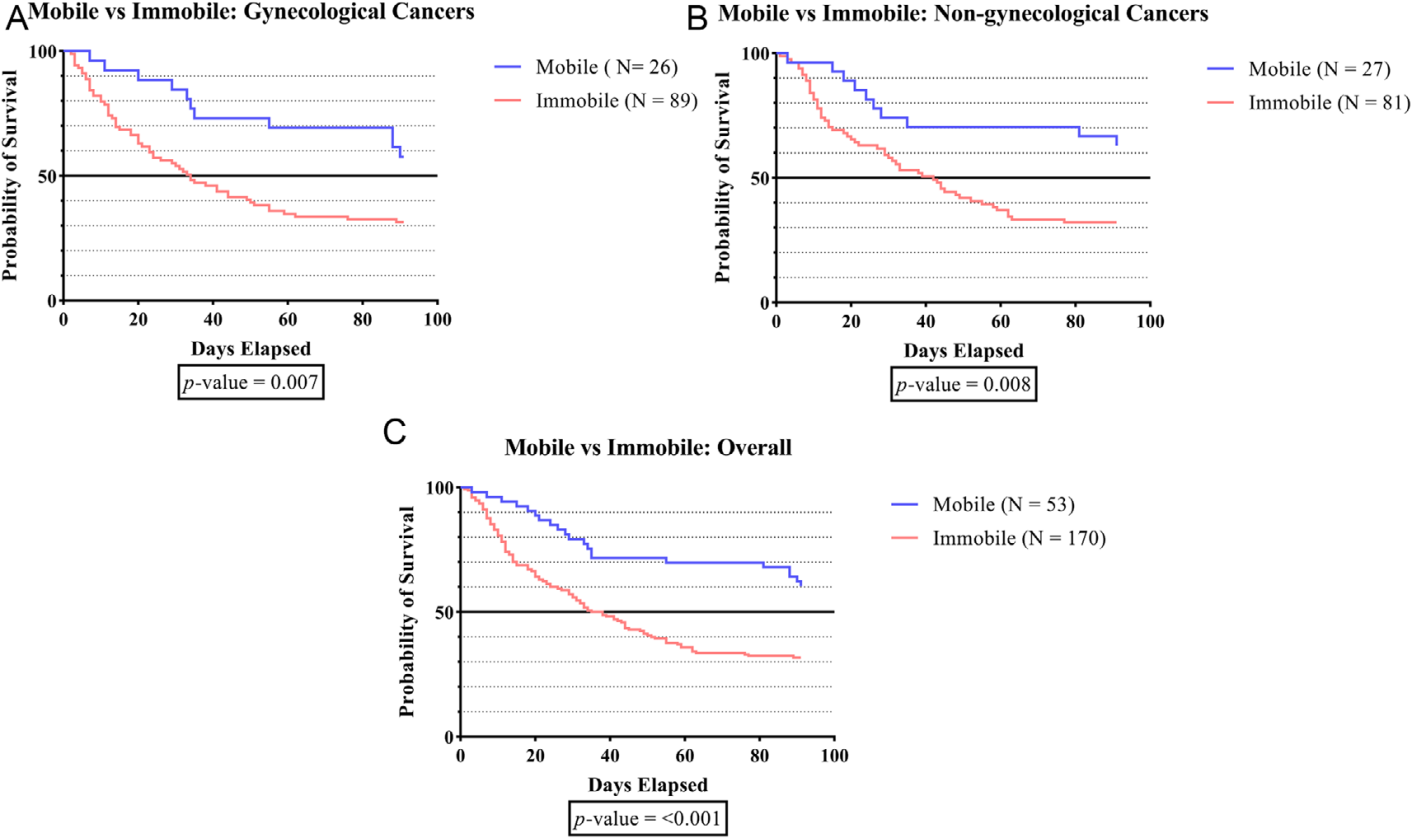
The survivability comparison of cancer patients with deep vein thrombosis among different immobilization status. Analyzed using Kaplan-Meier survival analysis, significance was measured using log-rank/Mantel-Cox test.

Figure 1B indicates that, across all cancer groups, the survival duration was significantly shorter in late-stage cancer than in early-stage cancer (p-value <0.045).

**Figure 1B.**
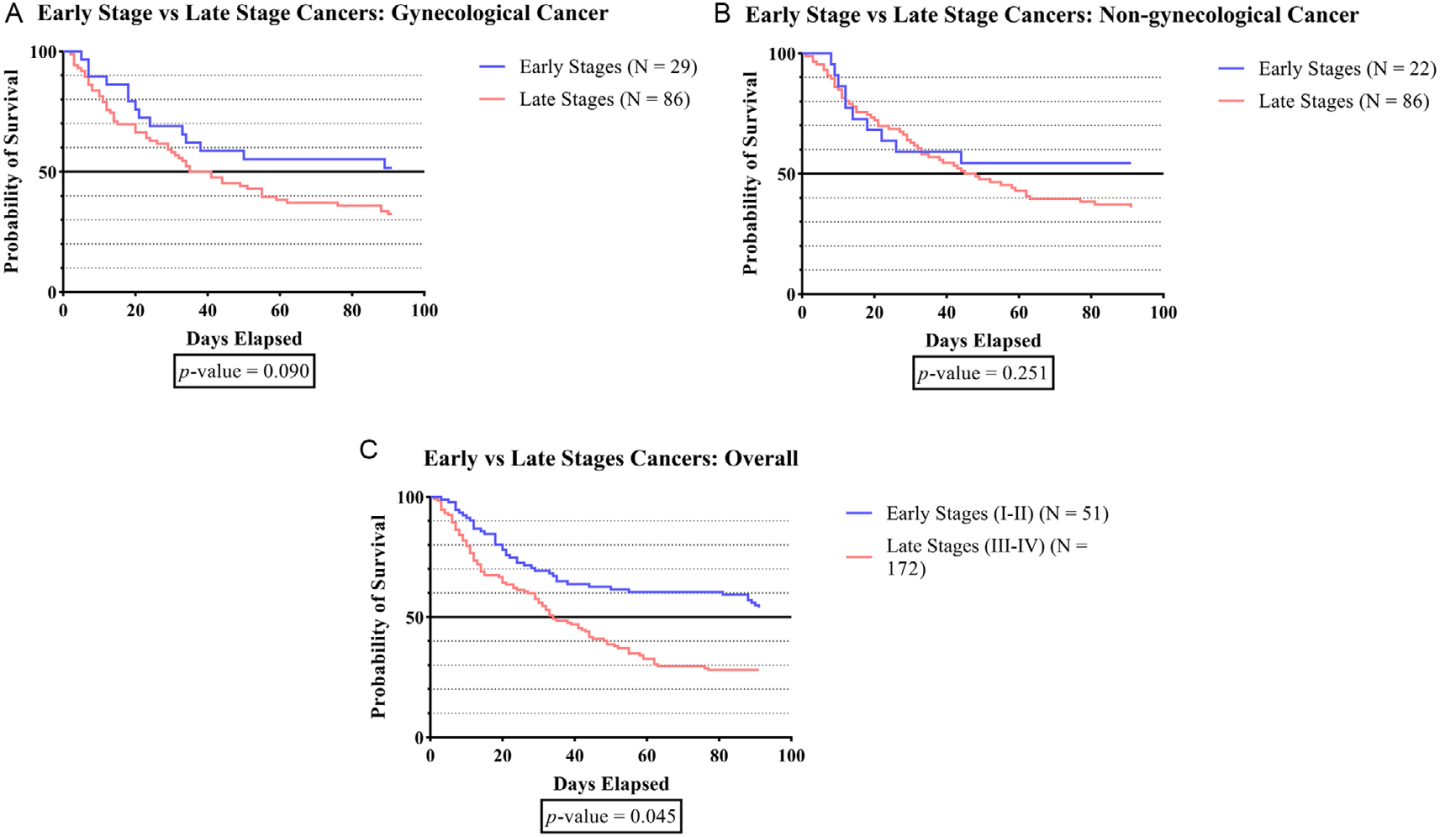
The survivability comparison of cancer patients with deep vein thrombosis among different cancer stages. Analyzed using Kaplan-Meier survival analysis, significance was measured using log-rank/Mantel-Cox test.

As seen in Figure 1C, subjects with infection status had significantly shorter survival times than subjects without infection in all categories (p-value 0.001), those with gynecologic cancer (p-value 0.021), and those without gynecologic cancer (p-value 0.002).

**Figure 1C.**
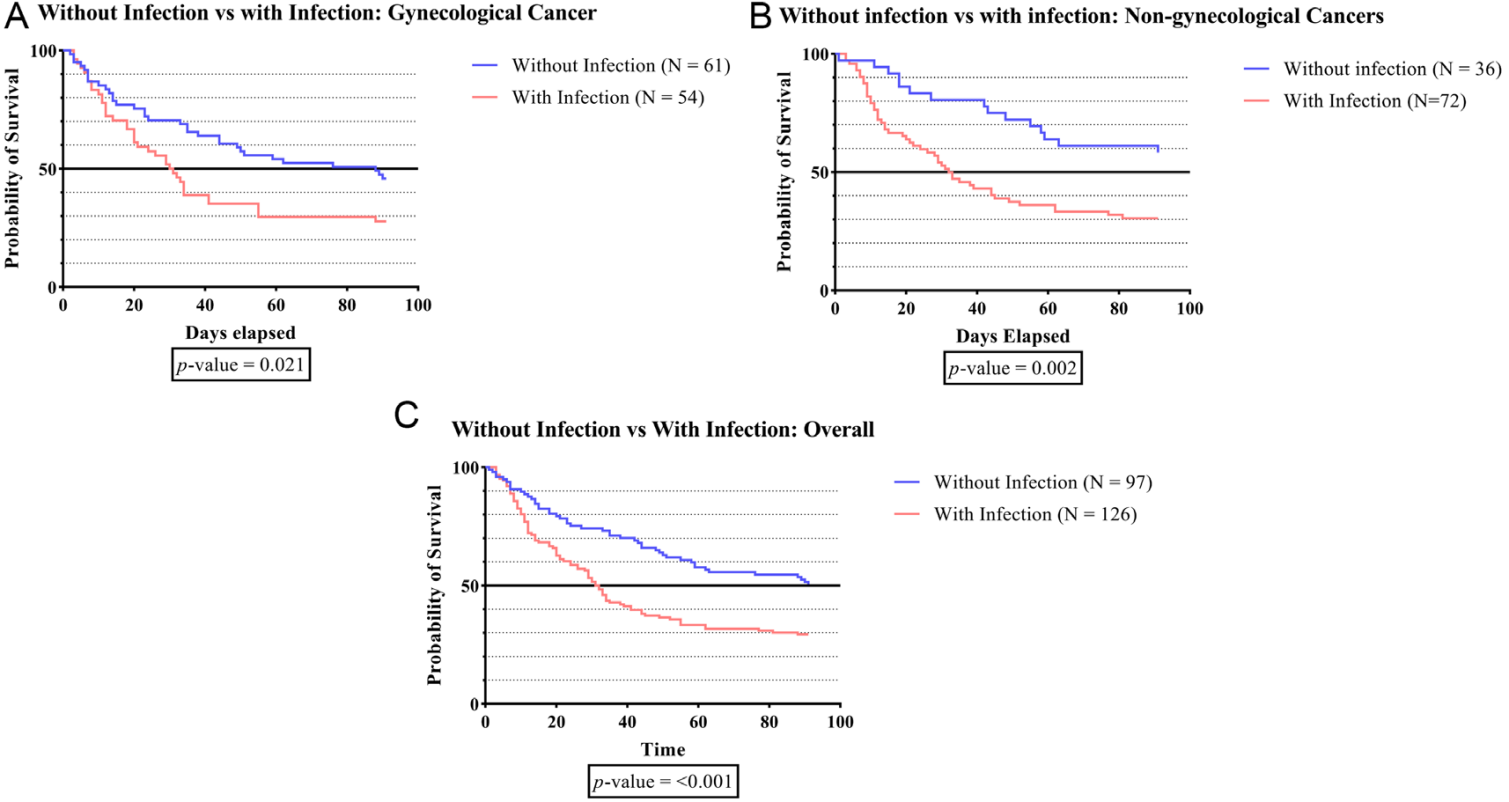
The survivability comparison of cancer patients with deep vein thrombosis among different infection status. Analyzed using Kaplan-Meier survival analysis, significance was measured using log-rank/Mantel-Cox test.

In the non-gynecologic cancer group shown in Figure 1D, the survival time was significantly longer in subjects who underwent cancer surgery than in those who did not (p-value 0.024).

**Figure 1D.**
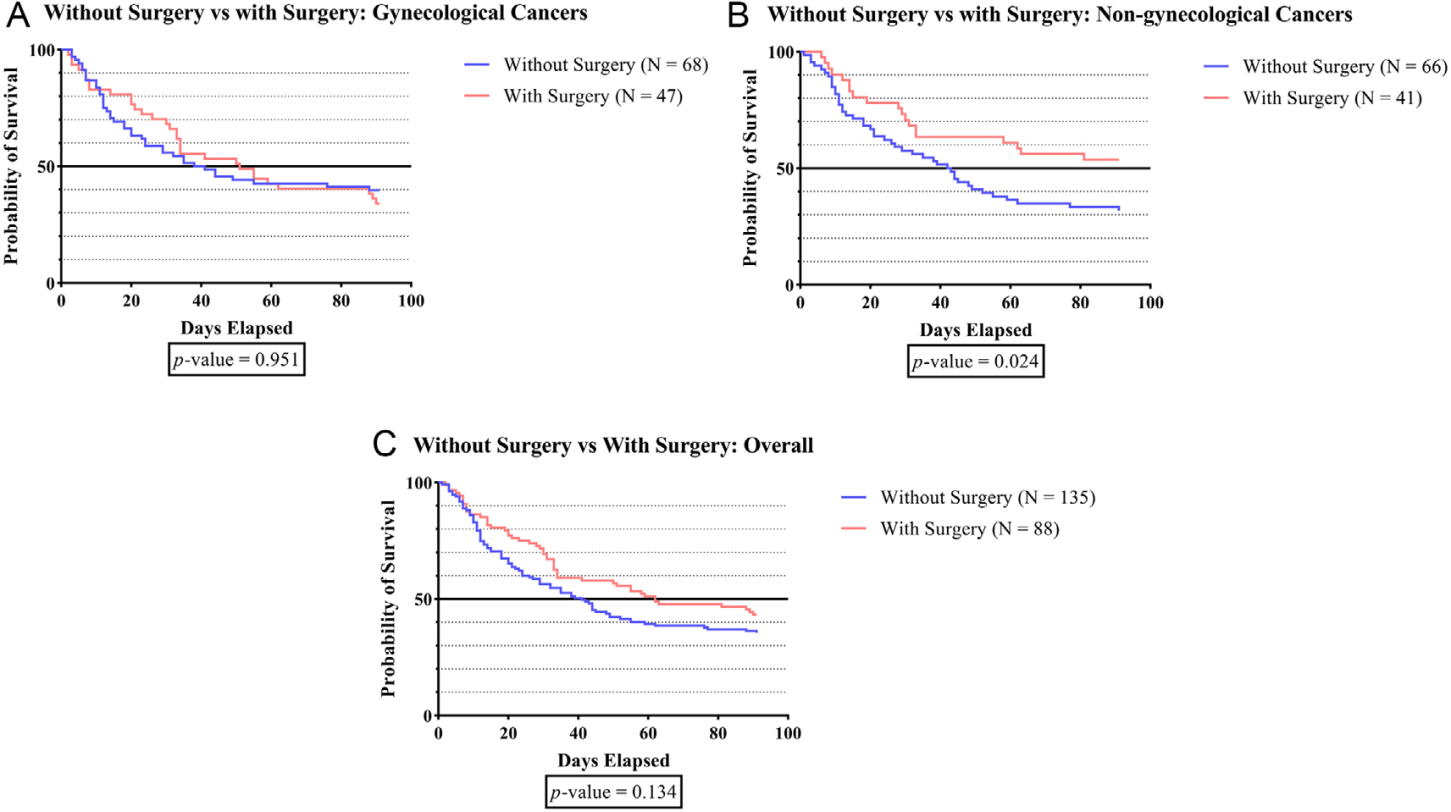
The survivability comparison of cancer patients with deep vein thrombosis among different cancer surgery status. Analyzed using Kaplan-Meier survival analysis, significance was measured using log-rank/Mantel-Cox test.

Figure 1E demonstrates that there was no significant survival time difference between subjects who received radiotherapy (RT) and those without RT in all groups.

**Figure 1E.**
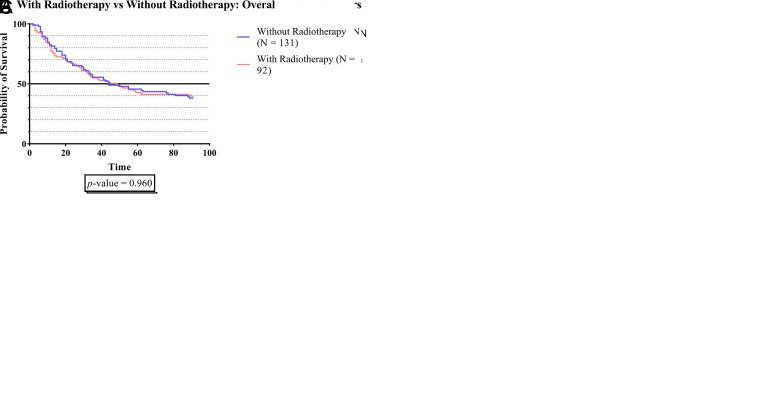
The survivability comparison of cancer patients with deep vein thrombosis among different radiotherapy status. Analyzed using Kaplan-Meier survival analysis, significance was measured using log-rank/Mantel-Cox test.

Figure 1F shows that in the non-gynecologic cancer group, subjects who received high-risk systemic therapy had significantly greater survival times than those who received low-risk systemic therapy (p = 0.048).

**Figure 1F.**
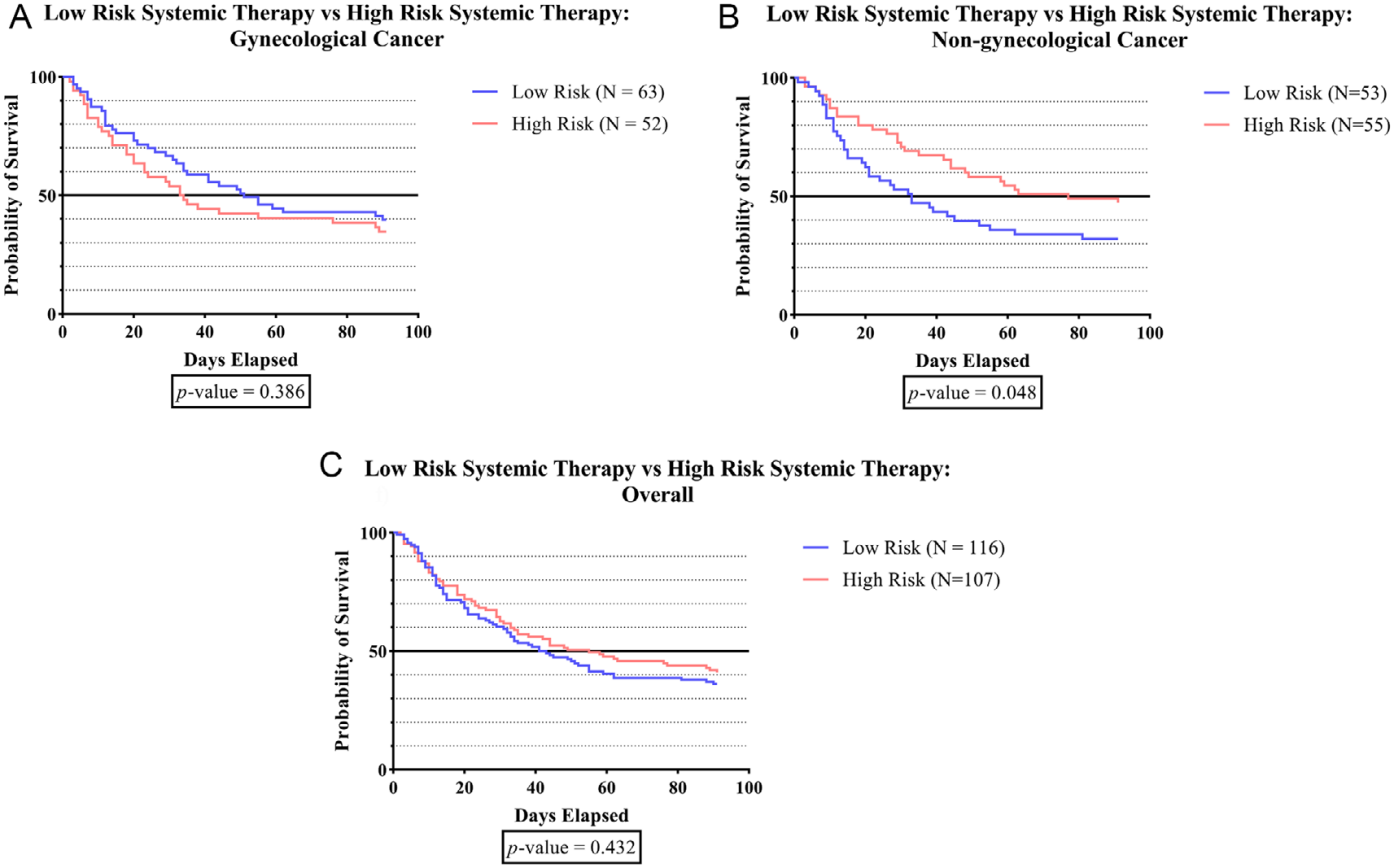
The survivability comparison of cancer patients with deep vein thrombosis among different systemic therapy status. Analyzed using Kaplan-Meier survival analysis, significance was measured using log-rank/Mantel-Cox test.

**Figure 2.**
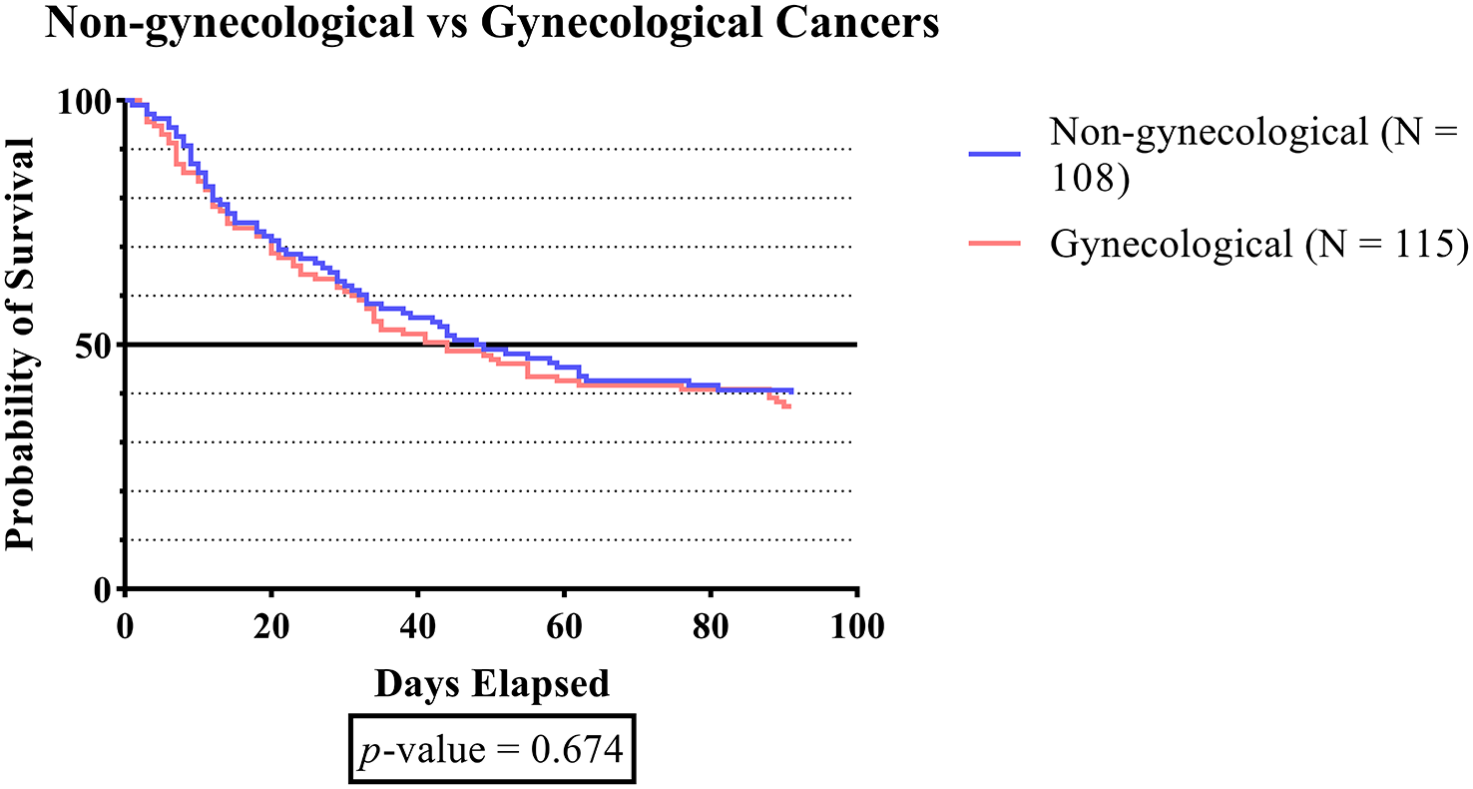
The survival plot of subjects with gynecologic cancers vs. subjects without gynecologic cancers.

Figure 2 demonstrates that although patients with gynecologic cancer generally had shorter survival times than those with non-gynecologic cancer, there was no significant difference in survival times between the two types of cancer.

## Discussion

Deep vein thrombosis (DVT) in cancer patients is a life-threatening condition that may emerge regardless of whether they have a good prognosis. By understanding the factors that affect survivability, the effectiveness of prophylactic and therapeutic measures in preventing mortality can be more fully established. ^23^

Our cohort analysis revealed that the mortality of gynecologic and non-gynecologic cancer patients doubled during the initial 3-month period following the DVT event, emphasizing the high short-term mortality resulting from a DVT event ( Table 1). ^23^^,^^24^ Our finding was supported by the worldwide *Registro Informatizado de la Enfermedad TromboEmbólica* (RIETE) registry study, which demonstrated that individuals with cancer with DVT are at a higher risk for short-term mortality. ^24^ The proposed theory suggests that the clinical hypercoagulable state may serve as a proxy for aggressive tumor biology, leading to a poor association with prognosis. ^24^^,^^43^ According to the latest available data, thrombosis is estimated to contribute to approximately 10% of cancer-related deaths in patients undergoing chemotherapy. Our study, which focused on gynecologic and non-gynecologic cancer patients with DVT, has confirmed this finding, albeit to a slightly greater degree. ^24^^,^^44^

Table 1 demonstrates a greater number of subjects experiencing immobilization (> 3 days) compared to those who did not (76.2% vs. 36.8%). The survival rate was significantly lower in subjects who developed immobilization status when compared to subjects without immobilization status in the overall groups (p-value <0.001), gynecologic cancer group (p-value 0.007), and non-gynecologic cancer group (p-value 0.008) ( Figure 1A). The lower survival time among immobilized subjects is a crucial finding that can be assigned to various factors. First, immobilized cancer patients frequently showed more severe clinical signs than non-immobilized patients. ^45^ Second, immobility increases the risk of DVT, due to stasis of blood flow in the venous system and hypercoagulation, which will lead to a higher mortality rate. ^46^^,^^47^ On the other hand, the American Physical Therapy Association (APTA) recommends that cancer patients with DVT commence mobilization as an effective way of reducing the risk of mortality and minimizing complications. ^48^^,^^49^ Another systematic review by Segal *et al.* that identified 2 guidelines, 18 systematic reviews, and 29 randomized controlled trials (RCT) demonstrates that exercise is safe and beneficial in improving quality of life, including muscular and aerobic fitness for cancer patients. The current literature provides adequate support for urging exercise among cancer patients. ^50^ Thus, we strongly recommend physical exercise based on the clinical condition of cancer patients, as it could potentially serve an essential function in improving both survival and quality of life.

Figure 1B shows that survival time was significantly lower in the advanced stages of cancer than in the early stages in the overall cancer groups (p-value <0.045). Our finding was consistent with a study that was conducted by Spencer, which found a negative correlation between cancer stage and survival. ^51^ Mark *et al.* found in their retrospective analysis that a diagnosis of DVT doubled the cancer-related death rate, which had similar effects on mortality as having advanced stage cancer. ^52^ Cancer staging is an anatomic representation of the disease’s progression based on tumor size, lymph node involvement, as well as clinical and imaging examinations. ^53^^,^^54^ The impact of metastases in DVT has been substantially highlighted in the medical literature. The process of disseminating metastatic cells could be the explanation for the increased risk of DVT reported in patients with distant or regional lymph node metastases. The presence of metastasis has been found to be significantly correlated with increased hypercoagulability due to the hemostatic system, which may play a significant role in the metastatic capability of malignancies. Substantial metastasis occurs when tumor cells reach the bloodstream or lymphatic system. Thereby, after the distant metastasis, tumor cells interact with the hemostatic system, which emphasizes that hypercoagulability already exists in patients with localized cancer spread. ^55^ Studies have shown that detecting cancer at an early stage can significantly improve the chances of survival. However, over 50% of cancer cases are diagnosed at an advanced stage. When diagnosed in later stages, the available treatment options may be limited, and the overall prognosis tends to be unfavorable. The timely identification of cancer or precancerous alterations enables prompt intervention with the purpose of preventing or minimizing cancer progression and mortality. ^56^

In this present study, pneumonia was the most prevalent infectious disease (29.6%) ( Table 2). In contrast with the study by Gussoni G, *et al.* that showed non-infectious chronic pulmonary disease contributed to the high prevalence of malignancy associated with DVT (9.8%). ^24^ This is due to the high prevalence of infectious diseases in Indonesia. The vast majority of infected patients have one source of infection (30.5%). This demonstrates that the severity of that illness has the potential to alter mortality, even if the patient is only exposed to a single source of infection. Figure 1C shows that the overall (p-value <0.001), gynecologic (p-value 0.021), and non-gynecologic (p-value 0.002) cancer groups had significantly shorter survival times for subjects who developed infection status compared to subjects without infection. Infection remains a leading killer in all types of cancer. The investigation of infection management in cancer patients is an important strategy for enhancing the patient’s survivability. The nature of certain chemotherapeutic agents is accompanied by their immunosuppressive effects, which can limit their application ranges. These side effects include neutropenia, inhibition of the neutrophils, bone marrow suppression, damage to the anatomical barriers, and irritation of the veins. ^57^ Those who undergo cytotoxic chemotherapy are at risk of developing severe neutropenia, which can lead to life-threatening infections and sepsis. ^58^ The findings indicate that further efforts are required to reduce the frequency of lethal infections. Thus, we strongly recommend several strategies to decrease the mortality of infections in cancer patients, including the use of prophylaxis or preemptive therapy with broad-spectrum antimicrobial agents targeted at the most common infecting pathogens. ^57^

Figure 1D demonstrates that the survival time for patients with non-gynecologic cancer who received cancer surgery is considerably longer than that of patients who do not undergo surgery (p-value 0.024). The present study aligns with prior cohort study of advanced stage non-small cell lung cancer (NSCLC) patients from the National Cancer Database, which indicated patients who had surgery had significantly better overall survival (p<0.001). ^59^ Contrary to the findings of Gussoni G, *et al.* in the RIETE Study, surgical treatment for malignancy had no influence on mortality in the first three months of malignancy DVT. ^24^ These differences in outcomes may be explained due to the fact that the patients who received surgery tended to be diagnosed at an earlier stage, which enhanced their chances of survival. As the advanced stage of cancer progresses, the clinical condition of the patient deteriorates, rendering them ineligible for cancer surgery and/or systemic therapy for malignancy. In the execution of a surgical procedure, it is imperative to carefully evaluate the potential hazards and advantages. This critical assessment is crucial in ensuring the safety and well-being of the patient. The risk-benefit analysis is the key of clinical decision making, with the ultimate goal of producing the best possible patient outcomes. ^60^

In Figure 1E, there was no significant difference in survival time between subjects who received RT and without RT across all groups. Based on our findings, we identify that RT-treated cancer patients with DVT do not appear to have substantial alterations that lead to short-term mortality. Our study was in line with Bosco *et al.* that conducted a large study among 9000 Swedish male patients who had curative radiation for prostate cancer. They concluded that external beam radiation and brachytherapy were not shown to increase the risk of thromboembolic events. ^61^^,^^62^ Contradicting the results of a previous sub-analysis of prospective, multicentre, longitudinal Prospective Comparison of Methods for thromboembolic risk assessment with clinical Perceptions and AwareneSS in real life patients-Cancer Associated Thrombosis (COMPASS–CAT study), a significant association was found between RT and VTE (HR 2.47; 95% CI 1.47-4.12; p-value 0.011). ^61^^,^^63^ A small series by Guy *et al.* reported a sufficient association between brachytherapy and the development of VTE in patients with gynecologic malignancies. ^63^^,^^64^ These differences among studies might be explained by multiple suggested processes that raised the risk of thrombosis during and after radiation. Radiation has been shown to promote secondary venous hemostasis by increasing inflammatory molecule release, which in turn activates the endothelium and promotes a thrombotic environment. Ionizing radiation has also been shown to affect several anticoagulant molecules (protein C and thrombomodulin) and prothrombotic molecules (activated factor VIII, platelet, tissue factor, D-dimers, NF-kappa B, and von Willebrand activation), altering the balance towards a hypercoagulable state. ^61^^,^^62^^,^^65^ However, most of the chronic vascular processes leading to clinical outcomes are reported to occur several years after RT, and our follow-up period was 3 months. Thus, additional studies with longer time periods are warranted to fully comprehend the clinical significance of the prospective cellular toxicity related to radiotherapy. ^61^^,^^62^

As can be observed in Figure 1F, the survival time was significantly higher in subjects who received high-risk systemic therapy compared to those who received low-risk systemic therapy (p-value 0.048). According to recent research, the administration of tamoxifen, aromatase inhibitors, thalidomide, lenalidomide, bevacizumab, cisplatin, nitrogen mustard, or anthracycline has been linked to a higher incidence of DVT. ^1^^,^^26^^–^^41^ These agents have been reported to initiate vascular injury by inducing apoptosis. The administration of cisplatin leads to the release of prothrombotic particles, leading to the production of thrombin through tissue factor-independent pathways and a substantial increase in the activity of von Willebrand factor (vWF). However, other agent such as the vascular endothelial growth factor inhibitor (bevacizumab) do not initiate thrombosis directly. Instead, it ‘primes’ the endothelium by placing it in a depleted condition, thus rendering it more prone to injury. Furthermore, in the context of immunomodulatory agents (thalidomide and lenalidomide), platelet activation via PAR-1 and enhanced Gp IIb/IIIa exertion provide the “primed” condition. The identification of antineoplastic agents that are highly associated with thrombosis can enhance healthcare provider awareness and facilitate prompt diagnosis and treatment. ^66^^,^^67^ On the other hand, prior studies have extensively demonstrated the effectiveness of systemic therapy in improving overall survival rates and minimizing the potential of disease recurrence. Although generally well-tolerated, there have been reports of various adverse effects associated with the pharmacological activity of these agents. ^67^ Thus, high-risk systemic therapy for cancer patients should be considered after carefully assessing the risks and benefits of treatments. ^68^

To the best of our knowledge, no study to date has investigated the comparison of survival time for DVT in the cancer population, especially among gynecologic and non-gynecologic malignancies. Although the difference in survival time between the two groups was not statistically significant (p-value 0.674), the results indicated that patients with gynecologic cancer generally had a lower survival time compared to non-gynecologic cancer ( Figure 2). Cancer is a life-threatening disease that warrants serious attention, regardless of its type. ^69^ According to prior research, VTE is a significant cause of mortality in patients with gynecologic cancer. Studies have shown that the risk of DVT in women undergoing gynecologic surgery is estimated to be between 17-40%. ^55^ The relationship between gynecologic cancers and thrombotic events is believed to be linked to lymphadenectomy and venous congestion caused by tumors or enlarged lymph nodes. ^70^ Lymphadenectomy is a frequently utilized procedure in the evaluation of lymph node status and staging of gynecologic malignancies, as well as a therapeutic intervention for patients with gynecologic cancer. Complications, such as hemorrhage, hematoma, and lymphocele, are frequently observed in association with it. Lymphocele is a frequently observed postoperative complication that can result in the development of VTE due to venous compression. A literature review indicates that the incidence of VTE after lymphadenectomy ranges from 0.8% to 25%. ^55^ A large cohort study conducted by Chew *et al.*, revealed that the incidence of DVT in cancer patients is linked to a 3.7-fold rise in mortality risk. ^71^ The identification of DVT as a major risk factor has been established for decreased survival in cancer patients. The mortality rate of cancer patients with DVT could be associated with the existence of DVT and its complications, as well as the advanced stage of malignancies that are frequently correlated with DVT and have the potential to progress progressively. ^24^

The strength of this study lies in the fact that it is the first study to analyze factors affecting the survivability of malignant patients with DVT, especially in gynecologic and non-gynecologic malignancies. Second, this study utilizes a pragmatic approach by conducting research in authentic settings that reflect routine clinical practice. Third, this research was conducted at CMGH, which is the national referral center hospital in Indonesia, in order to accurately reflect the target population. However, this study has several limitations that should be considered to improve further research. First, this study did not evaluate the development of anticoagulant targets in DVT therapy in patients with malignancy. The examination parameters for each anticoagulant vary, making it challenging to perform this task. Second, this study did not differentiate between varying levels of clinical severity of infection.

Hence, despite these limitations, the findings of our cohort study have significant clinical implications, indicating that prompt identification of these factors may enhance the survival rate of cancer patients with DVT. ^72^ Although cancer is a significant risk factor for DVT, the risk is not sufficiently substantial to warrant initiating prophylaxis. On the other hand, if any of these risk factors are present, the doctor should have a low threshold for considering prophylaxis. ^73^ Comprehensive consideration of the risks and benefits of anticoagulant usage is required before deciding to use thromboprophylaxis in cancer patients with DVT. ^68^ Nonetheless, the existing literature provides limited guidance for decision-making and implementation of management strategies. Further studies are warranted to evaluate the extent of the issue and determine the necessity of secondary prophylaxis. ^24^^,^^73^

## Conclusion

This cohort study identified that both gynecologic and non-gynecologic cancer patients who experienced DVT developed a high short-term mortality. Our finding of immobility, infection, advanced cancer stages, systemic therapy, and cancer surgery as risk factors that affect the survival raises the need to give secondary prophylaxis in routine clinical practice. The possibility for increasing survival could be maximized through the alteration of these critical factors. Thus, further study is warranted to establish a comprehensive management guideline that will be crucial for optimizing prevention and treatment strategies.

## Data Availability

Figshare: Raw Data - The Factors Affecting the Survivability of Malignant Cancer Patients with Deep Vein Thrombosis among Subjects with Gynecologic and Non Gynecologic Cancer An Ambispective Cohort Study.sav, https://doi.org/10.6084/m9.figshare.22838087.v1. ^74^ Data are available under the terms of the Creative Commons Zero “No rights reserved” data waiver (CC0 1.0 Public domain dedication).
