# Supplementary material for: The factors affecting the survivability of malignant cancer patients with deep vein thrombosis among subjects with gynecologic and non-gynecologic cancer: An ambispective cohort study
Source: F1000Res. 2023 Dec 12;12:890. Originally published 2023 Jul 26. [Version 2] doi: 10.12688/f1000research.135252.2 (PMC11522705; doi:10.12688/f1000research.135252.2)
Supplement: Peer review history

# Reviewer report: Reviewer response for version 2

**DOI:** 10.5256/f1000research.155831.r328897

**Published:** 2024-10-29

**Authors:** Budi Setiawan

**Affiliations:** 1 Dr. Kariadi General Hospital, Semarang, Indonesia

This research’s main aim is to determine the factors affecting survival of gynecologic and non-gynecological cancer having DVT, but theoretically, most cancer patients with DVT have lower survival rate, regardless the diagnosis. Can you explain why did you need to analyze between gynecologic and non-gynecologic cancer, especially in your introduction?

 I suggested that hematologic malignancies should be excluded from analysis, as patient with hematologic malignancies had different characteristics compared with patient with solid tumors.

 Looking in your current result and conclusion, there was no significant differences in survival between gynecologic and non-gynecologic cancer having DVT. I think it is more suitable if this paper mainly discusses about factors affecting survival in cancer patients having DVT.

 Several concerns that needed to be clarified:

 Please mention your reason on choosing ambispective cohort study, as it can potentially lead into bias.

 Please provide the reason why did you not included central venous catheter, D-Dimer, and Well’s score in your analysis.

 Theoretically, is there any difference in DVT incidence between men and women? In non-gynecological group, you included both men and women, and if I assume that all of the patient having gynecological cancer is women, does that mean gender can be a confounding variable in this study?

 Please mention about infection and DVT in your introduction and method section, including how did you diagnose the infection status.

 Did the DVT diagnosis was made before or after receiving any therapy e.g. chemotherapy / radiotherapy?

Is the work clearly and accurately presented and does it cite the current literature?

Yes

If applicable, is the statistical analysis and its interpretation appropriate?

Yes

Are all the source data underlying the results available to ensure full reproducibility?

Partly

Is the study design appropriate and is the work technically sound?

Partly

Are the conclusions drawn adequately supported by the results?

Partly

Are sufficient details of methods and analysis provided to allow replication by others?

Partly

Reviewer Expertise:

thrombosis and hemostasis, hematology, medical oncology

I confirm that I have read this submission and believe that I have an appropriate level of expertise to confirm that it is of an acceptable scientific standard, however I have significant reservations, as outlined above.

# Reviewer report: Reviewer response for version 2

**DOI:** 10.5256/f1000research.155831.r305997

**Published:** 2024-08-29

**Authors:** Hanno Riess

**Affiliations:** 1 Charite - Universitatmedizin Berlin, Berlin, Germany

The authors make a long introduction, the study protocol is not innovative. The results present challenges in interpretation due to the statistical analysis conducted on a small, incompletely defined, and highly heterogeneous population.

 The "Results" confirm available evidence and than they add a very long discussion, in my view this needs further refinement to enhance its usefulness, as there are several well-done reviews dealing with DVT, stage, infection etc. in cancer patients.

 The authors present an investigation dealing with well accepted risk factors of death in cancer patients. Thus it is strongly recommended to rephrase the title, including "confirmatory study".

 The patient population is very heterogenous:  As cancer stage is a major determinator of prognosis it is recommended to add 2 additional columns in Table 1 giving the stage of the pts for each line.

 Several things need to be clarified: 

 - Is this a mixture of in- and out-pts ? Are pts from an palliative care unit ("hospice") included ?

 - With regard to radiation, surgery and chemotherapy, does that mean these treatment modalities have been applied prior to DVT diagnosis or thereafter or both ? 

 - What is meant with "immobilisation"? In clinical practice "immobilisation" is a very rare event! Thus, please give a definition of "immobilisation" such as for example "reduced mobility with bedrest time about > 70 % of the day". 

 - The Wells score has limited applicability for prediction of outcome after DVT. In pts with DVT Wells score is expected to be high in most cancer pts (cancer is one parameter in this score) as reflected in table 1. Overall it is not a useful parameter in this setting, thus it is recommended to omit it. 

 - "Mortality doubled"  compared to ? (p10, last paragraph)

 I suggest the authors to reduce Introduction and Discussion (there are lots of reviews on VTE in cancer and additional risk factors beside cancer) and expand Limitations (no RCT, prospective & retrospective; patient heterogeneity, DVT treated yes or no, for three months?...)

Is the work clearly and accurately presented and does it cite the current literature?

Partly

If applicable, is the statistical analysis and its interpretation appropriate?

Partly

Are all the source data underlying the results available to ensure full reproducibility?

No

Is the study design appropriate and is the work technically sound?

Partly

Are the conclusions drawn adequately supported by the results?

Partly

Are sufficient details of methods and analysis provided to allow replication by others?

No

Reviewer Expertise:

oncology, hematology, hemostaseology; GI-cancers, venous thromboembolism (VTE), cancer-associated VTE.

I confirm that I have read this submission and believe that I have an appropriate level of expertise to confirm that it is of an acceptable scientific standard, however I have significant reservations, as outlined above.

# Reviewer report: Reviewer response for version 1

**DOI:** 10.5256/f1000research.148362.r195071

**Published:** 2023-09-01

**Authors:** Susanna Hutajulu, Juan Wiranata

**Affiliations:** 1 Division of Hematology and Medical Oncology, Department of Internal Medicine, Faculty of Medicine, Public Health and Nursing, Dr Sardjito General Hospital,, Universitas Gadjah Mada, Yogyakarta, Special Region of Yogyakarta, Indonesia; 2 Faculty of Medicine, Gadjah Mada University, Yogyakarta, Special Region of Yogyakarta, Indonesia

This study aimed to analyze the factors influencing the short-term survival (3 months) of cancer patients with DVT, particularly among those with gynecologic and non-gynecologic cancers. Employing an ambispective cohort study design, a total of 223 cancer patients diagnosed with DVT via Doppler ultrasound were included in this study.

 The findings revealed that, across the entire cohort, survival time was notably reduced among subjects who experienced immobilization, were at advanced cancer stages, or developed infections. Within the gynecologic cancer subgroup, survival time was significantly lower in individuals who experienced immobilization and infection. Conversely, within the non-gynecologic cancer subgroup, survival time exhibited a significant decline in subjects who faced immobilization, infection, cancer-related surgeries, and high-risk systemic therapy for DVT.

 This study presents novel evidence for the local population, underscoring the significance of both preventing and managing DVT in cancer patients.

 There are certain concerns that the authors might need to take into consideration:

- The method section of abstract is not so clear. I’m sure it needs brief details of methods applied in the study.
- The content within the second and third paragraphs of the results section, which elucidates the classification of DVT risk based on the chemotherapy regimen and provides an explanation of the Wells score, would be better placed within the method section. This relocation would contribute to a comprehensive depiction of the operational definition of the variables.
- Proportion of radiotherapy utilization can be incorporated into Table 1, as this aspect has not yet been delineated within the existing presentation of the table.
- To enhance clarity, it would be better to add the "number at risk" and "number censored" below each individual point segment of the survival curve.

Is the work clearly and accurately presented and does it cite the current literature?

Yes

If applicable, is the statistical analysis and its interpretation appropriate?

Yes

Are all the source data underlying the results available to ensure full reproducibility?

Yes

Is the study design appropriate and is the work technically sound?

Yes

Are the conclusions drawn adequately supported by the results?

Yes

Are sufficient details of methods and analysis provided to allow replication by others?

Partly

Reviewer Expertise:

Clinical oncology, cancer prognostication, cancer registry

We confirm that we have read this submission and believe that we have an appropriate level of expertise to confirm that it is of an acceptable scientific standard.

# Response

**Published:** 2023-09-08

**Authors:** Andhika Rachman

**Affiliations:** Internal Medicine, Universitas Indonesia, Jakarta, DKI Jaya, Indonesia

**Response To The Reviewer:**

 Dear Dr. Susanna Hutajulu,

 We would like to thank you for your time and consideration in handling our manuscript.

 Sincerely,

 Andhika Rachman, MD, PhD

 Medical staff, Division of Hematology and Medical Oncology, Department of Internal Medicine, Faculty of Medicine, Universitas Indonesia - Dr. Cipto Mangunkusumo General Hospital

 This study aimed to analyze the factors influencing the short-term survival (3 months) of cancer patients with DVT, particularly among those with gynecologic and non-gynecologic cancers. Employing an ambispective cohort study design, a total of 223 cancer patients diagnosed with DVT via Doppler ultrasound were included in this study.

 The findings revealed that, across the entire cohort, survival time was notably reduced among subjects who experienced immobilization, were at advanced cancer stages, or developed infections. Within the gynecologic cancer subgroup, survival time was significantly lower in individuals who experienced immobilization and infection. Conversely, within the non-gynecologic cancer subgroup, survival time exhibited a significant decline in subjects who faced immobilization, infection, cancer-related surgeries, and high-risk systemic therapy for DVT.

 This study presents novel evidence for the local population, underscoring the significance of both preventing and managing DVT in cancer patients.

 There are certain concerns that the authors might need to take into consideration:

**1. **The method section of abstract is not so clear. I’m sure it needs brief details of methods applied in the study.  

**Response:**

 Thank you for your constructive comment. We have added more details on the method section of the abstract.

 Methods: An ambispective cohort study was conducted among gynecologic and non-gynecologic cancer patients with DVT, from January 2011 until August 2013. All subjects were observed for three months. The presence of DVT was confirmed using Doppler ultrasound. The analysis was performed using Kaplan-Meier survival analysis. The statistical significance was determined using the log-rank/Mantel-Cox test.

**2. **The content within the second and third paragraphs of the results section, which elucidates the classification of DVT risk based on the chemotherapy regimen and provides an explanation of the Wells score, would be better placed within the method section. This relocation would contribute to a comprehensive depiction of the operational definition of the variables.  

**Response:**

 We have relocated the second and third paragraphs of the results section into the method section.

 Wells score has been utilized for over a decade and has predictive value in determining DVT risk in patients who are hospitalized. ^22^^ ,^^42^ We divided the Wells score into two categories: subjects with <3 points were considered to have a low probability of developing DVT, whereas subjects with ≥3 points were considered to have a high probability of developing DVT.

 Subjects who received tamoxifen, aromatase inhibitors, thalidomide, lenalidomide, bevacizumab, cisplatin, nitrogen mustard, or anthracycline were considered as high-risk systemic therapy for developing DVT. ^1^^ ,^^26^^ –^^41^Subjects who did not receive high-risk systemic therapy other than those mentioned above were considered low-risk for developing DVT.

**3. **Proportion of radiotherapy utilization can be incorporated into Table 1, as this aspect has not yet been delineated within the existing presentation of the table.  

**Response:**

 We have added the proportion of radiotherapy utilization into Table 1.

**4. **To enhance clarity, it would be better to add the "number at risk" and "number censored" below each individual point segment of the survival curve.

**Response:**

 Thank you for your valuable advice. We have added the “number at risk” below each individual point segment of the survival curve.

 Thank you for your attention. We are eagerly awaiting your response.
